# Differences in the Relative Abundance of ProBDNF and Mature BDNF in A549 and H1299 Human Lung Cancer Cell Media

**DOI:** 10.3390/ijms22137059

**Published:** 2021-06-30

**Authors:** Sadaf Dorandish, Sarah Atali, Ravel Ray, Hind Al Khashali, Kai-Ling Coleman, Jeffrey Guthrie, Deborah Heyl, Hedeel Guy Evans

**Affiliations:** Chemistry Department, Eastern Michigan University, Ypsilanti, MI 48197, USA; sdorandi@emich.edu (S.D.); satali@emich.edu (S.A.); rray9@emich.edu (R.R.); halkhash@emich.edu (H.A.K.); kcolem24@emich.edu (K.-L.C.); jguthri7@emich.edu (J.G.); dheylcle@emich.edu (D.H.)

**Keywords:** brain-derived neurotrophic factor, lung cancer, matrix metalloproteinase, p53, PI3K, AKT, NFκB

## Abstract

Brain-derived neurotrophic factor (BDNF), a member of the neurotrophin family, has been linked to several human malignancies and shown to promote tumorigenesis. The purpose of this study was to explore the relative abundance of pro-brain-derived neurotrophic factor (proBDNF) and mature BDNF (mBDNF) in A549 (p53 wild-type) and H1299 (p53-null) lung cancer cell media. Higher levels of proBDNF were detected in the media of A549 cells than in H1299 cell media. Using inhibitors, we found that the levels of proBDNF and mBDNF in the media are likely regulated by PI3K, AKT, and NFκB. However, the largest change in these levels resulted from MMP2/9 inhibition. Blocking p53 function in A549 cells resulted in increased mBDNF and decreased proBDNF, suggesting a role for p53 in regulating these levels. The ratio of proBDNF/mBDNF was not affected by MMP2 knockdown but increased in the media of both cell lines upon knockdown of MMP9. Downregulation of either MMP2 or MMP9 by siRNA showed that MMP9 siRNA treatment of either A549 or H1299 cells resulted in decreased cell viability and increased apoptosis, an effect diminished upon the same treatment with proBDNF immunodepleted media, suggesting that MMP9 regulates the cytotoxic effects induced by proBDNF in lung cancer cells.

## 1. Introduction

Non-small-cell lung carcinoma (NSCLC) consisting of adenocarcinoma, squamous-, and large-cell carcinoma accounts for ~80% of all lung cancer cases and, despite recent advances in drug development, remains highly resistant to current cancer therapeutics [1,2].

**Figure 1:**
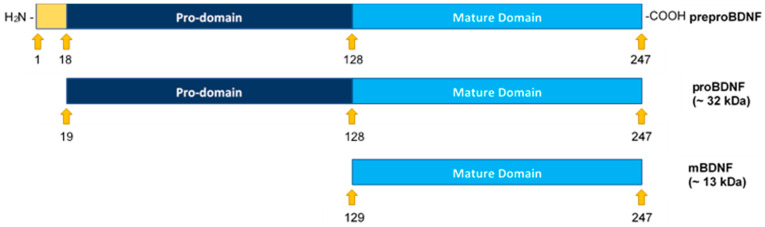
Structural representation of preproBDNF, proBDNF, and mature BDNF (mBDNF). Formation of mBDNF occurs upon cleavage of the N-terminus of proBDNF. The signal peptide (18 amino acids, positions 1–18) is shown in yellow, the propeptide (110 amino acids, positions 19–128) is shown in dark blue, and mBDNF (119 amino acids, positions 129–247) is shown in light blue.

Brain-derived neurotrophic factor (BDNF) (Figure 1), a member of the neurotrophin family of growth factors, and its high affinity primary receptor, tropomyosin receptor kinase B (TrkB), are widely known for the survival of neurons and synapses [3]. Over the years, however, many studies have highlighted the emerging functions of neurotrophins in a variety of cancers [3,4,5]. BDNF was shown to exert carcinogenic effects on different types of cancer and was reported to increase migration and survival of clear cell renal cell carcinoma, induce cell metastasis in human colon cancer, and promote proliferation and invasion of lung squamous cell carcinoma [6,7,8]. BDNF was found to be associated with poor prognosis in NSCLC patients, with levels upregulated in lung cancer cell culture supernatants compared with the normal lung counterparts and critical for lung tumorigenesis [7]. Tumor cells were reported to secrete BDNF, and both BDNF and TrkB have been shown to be upregulated in a wide variety of tumors [8]. With their recognized crucial role in the progression of cancer and as part of the superfamily of growth factor receptors with tyrosine kinase activities, the Trk receptors and their signaling pathways are suggested to constitute a therapeutic target for the development of anticancer drugs [6,8]. Involvement of BDNF in tumor pathogenesis through TrkB has been pointed out in different cancers including lung [7,8]. TrkB was found to be critical for lung cancer development, and its deficiency promoted apoptosis and significantly blocked metastasis of a lung adenocarcinoma model [7]. Expression of BDNF was reported to be moderate/weak in lung cancerous tissue but not detected in normal lung tissue, while expression of TrkB was found to be moderate/weak in lung cancerous tissue and moderate in normal lung tissue [6]. Silencing BDNF expression blocked cell proliferation and promoted cell apoptosis, thereby conferring a disadvantage to the growth of lung cancer cells [6,7,8,9].

All the neurotrophins are synthesized as proneurotrophin precursor forms including proBDNF, the precursor of BDNF [10]. Both BDNF and proBDNF are secreted with opposite functions on cell proliferation and apoptosis [6,8]. ProBDNF may be secreted extracellularly and cleaved by proteases such as matrix metallopeptidase 9 (MMP9) or cleaved intracellularly to yield C-terminal mature BDNF (mBDNF) (Figure 1) [3,8,10]. According to the literature, proneurotrophins are thought to trigger cell death whereas mature neurotrophins promote cell survival [9]. Unlike Trk receptors, p75^NTR^, the high affinity receptor for proneurotrophins, expressed in a wide range of human tumors, acts as a tumor suppressor and blocks cell proliferation and invasion in a variety of cancers, promoting good prognosis [9]. Using C6 glioma cells, proBDNF via p75^NTR^ led to increased apoptosis and reduced cell growth while a decreased proBDNF/mBDNF ratio was found in high-grade glioma and correlated negatively with tumor grade [11].

Upon binding of mBDNF to its receptor, a series of downstream pathways are stimulated, including the phosphoinositide 3 Kinase (PI3K)/AKT pathway, inducing oncogenic effects by promoting cell growth and survival [8]. PI3K is a heterodimer of a p85 regulatory subunit and a p110 catalytic subunit and major signaling molecule located downstream of growth factor receptor tyrosine kinases (RTKs) [12]. At the cell membrane, PI3K catalyzes the production of the lipid second messenger phosphatidylinositol-3,4,5-triphosphate (PIP3) resulting in recruitment and activation of downstream signaling components including the serine/threonine protein kinase, AKT [12,13]. Dysregulation of the PI3K/AKT signaling pathway, known to be associated with a number of human cancers, results in aberrant activation of numerous protein targets regulating a wide range of cellular processes critical for proliferation, tumorigenesis, survival, and growth [12,14].

The transcription factor and tumor suppressor protein, p53, is highly inducible by a range of stress signals, leading to regulation of the expression of a variety of genes that ultimately mediate the p53 response, including those involved in cell cycle arrest, apoptosis, senescence, and blocking cell proliferation [15,16]. Activation of p53 is known to contribute to apoptosis by inhibition of PI3K/AKT and nuclear factor kappa B (NFκB) [17,18]. Both p53 and NFκB are known to suppress each other’s ability to enhance gene expression [18]. In unstimulated cells, NFκB is bound to inhibitory IκB proteins, is inactive, and sequestered in the cytoplasm [19]. Upon stimulation, AKT induced phosphorylation results in degradation of IκB proteins, releasing NFκB which can then be translocated into the nucleus activating transcription [13,19,20]. Tumorigenicity was shown to be decreased upon blocking the activity of NFκB [19].

AKT was reported to promote the activity of NFκB, known to regulate the transcription of matrix metalloproteinases, MMP2/9, [13,21] members of the large family of MMPs, a group of Zn^2+^-dependent endopeptidases involved in degradation of extracellular matrix components and tumor progression [22]. MMPs are normally synthesized as inactive proenzymes that can become fully activated upon proteolytic processing [23,24]. MMP2 and MMP9 in particular among the MMPs are considered to be highly valuable enzymes due to the important role they play in disease pathogenesis such as cancer and Alzheimer’s disease [25]. Progression of different types of cancer including lung cancer has been reported to be associated with overexpression of both MMP2 and MMP9 and correlates with metastasis and poor prognosis [24,25,26]. A significant increase was reported for the expression of MMP2 and MMP9 in NSCLC compared with normal tissue and associated with invasion and metastasis [27,28]. MMP9 expression was reported to increase with tumor size and be significantly higher in metastatic NSCLC cases than in cases without metastasis [26]. Similarly, MMP2 expression in lung cancer tissues was shown to be higher than that found in normal tissues and suggested to be related to the development of lung cancer [29].

The MMP2 promoter is known to be regulated by p53 [30]. Overexpression of p53 was found to significantly inhibit MMP2 mRNA levels in several cell lines [31]. MMP9 promoter activity, along with mRNA and protein levels, was decreased by p53 in human soft tissue sarcoma, resulting in decreased tumor growth and cell invasion [32]. p53 was reported to result in decreased NFκB activity, and mutating the NFκB site in the MMP9 promoter eliminated the repressive effects induced by p53 suggesting a role for p53 in blocking MMP9 transcription via a mechanism that involves blocking activation of the MMP9 promoter by NFκB [32].

Based on these reports, we used two human NSCLC cell lines, [33] A549 (p53-positive) and H1299 (p53-null) [34] in this study to examine regulation of proBDNF and mBDNF levels in the media of lung cancer cells. We hypothesized that downregulating signaling of PI3K/AKT, NFκB, and MMP2/9 can result in increased extracellular proBDNF concentrations leading to cytotoxic effects on lung cancer cells via a mechanism involving p53.

## 2. Results

### 2.1. Higher Levels of ProBDNF Are Detected in the Media of A549 Cells than in H1299 Cell Media

Evidence has rapidly accumulated over the years highlighting how neurotrophins, including BDNF, play a role in cancer and their influence on cell growth and survival [4,5,6,8,9,35,36]. Compared to normal lung cells, the levels of BDNF were reported to be upregulated in lung cancer cell culture supernatants and critical for lung tumorigenesis [7]. TrkB was shown to be constitutively activated in human lung cancers and expressed at higher levels in tumor samples than in normal controls [35,37,38]. Moreover, in both A549 and H1299 cells, expression and activation of TrkB along with secreted BDNF in the media of A549 (26.6 ng/mL) and H1299 (63.2 ng/mL) cells, were reported [35].

**Figure 2:**
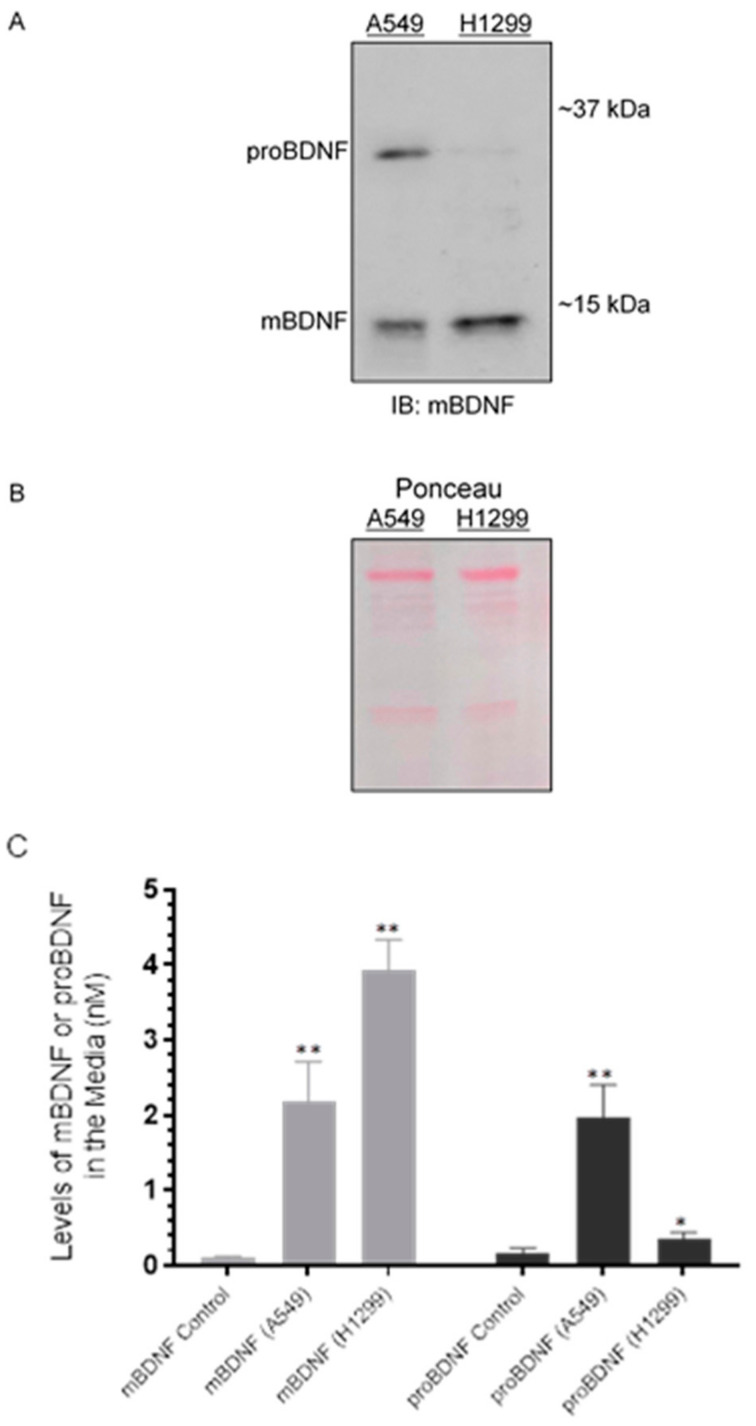
The levels of proBDNF are higher in the media of A549 cells than in the media of H1299 cells. Cells (0.2 × 10^5^) were grown in 10% FBS-supplemented media for 24 h in 25 cm^2^ flasks. The following day, the cell monolayers were incubated at 37 °C in serum-free media for 72 h. The same concentration of total protein (15 µL of 600 µg/mL) of the media (**A**) was used for Western blotting using the indicated antibody (IB: mBDNF). Total protein (Ponceau staining) (**B**) served as a loading control. The levels of mBDNF and proBDNF were quantitated as described in the Methods Section (**C**). Data from five independent assays, each carried out in triplicate, were quantitated and averaged using the GraphPad 9.0.2 software. The graph summarizes the results expressed as means ± SD (*n* = 5). Asterisks (*) indicate a statistically significant difference from the corresponding mBDNF or proBDNF control that included all components but using media not incubated with cells (C). * *p* < 0.05, ** *p* < 0.01, Mann–Whitney test.

We, therefore, tested whether there are differences in the levels of mBDNF and proBDNF in the media of A549 (p53-positive) and H1299 (p53-null) cells. Cells (0.2 × 10^5^) were grown in 10% FBS-supplemented media for 24 h followed by incubation at 37 °C in serum-free media for 72 h. The same concentration of total protein of the media was used for Western blotting using anti-mBDNF antibodies (Figure 2A,B). The levels of mBDNF and proBDNF were also quantitated (Figure 2C) as described in the Methods Section. Two bands were recognized in the media of A549 cells (Figure 2A) by Western blotting corresponding to the expected molecular weights of proBDNF and mBDNF, while one band was primarily detected in the media of H1299 cells migrating with the expected mBDNF molecular weight. Quantitation of mBDNF (Methods), using an anti-mBDNF capture antibody and a biotinylated anti-mBDNF detection antibody, showed that mBDNF levels are higher in H1299 cell media (~3.95 nM) compared to the media of A549 cells (~2.15 nM) (Figure 2C). Conversely, quantitation of proBDNF, using an anti-proBDNF capture antibody and a biotinylated anti-mBDNF detection antibody (Methods), showed higher levels of proBDNF in the media of A549 cells (~2.00 nM) as compared to that of H1299 cells (~0.35 nM) (Figure 2C). When the relative abundance of proBDNF and mBDNF was expressed as a ratio of the total BDNF signal (sum of proBDNF and mBDNF), mBDNF and proBDNF represented ~50% of the total in A549 cell media while ~92% and 8% were measured for mBDNF and proBDNF, respectively, in the media of H1299 cells. The ratio of proBDNF/mBDNF in the media of A549 cells was found to be ~1, while that in H1299 cell media was ~0.09. These results might suggest possible higher proteolytic cleavage of proBDNF to mBDNF in the media of H1299 cells.

### 2.2. Treatment of A549 and H1299 Cells with the MMP2/9 Inhibitor Resulted in Increased ProBDNF Levels and Corresponded with Decreased Levels of mBDNF in the Media, While the Opposite Effect Was Observed upon Treatment with the p53 Inhibitor, Pifithrin-α, in A549 Cell Media

We next attempted to identify key players involved in the signaling pathway leading to differences in proBDNF and mBDNF levels in the media of A549 and H1299 cells. Activation of PI3K/AKT signaling is known to regulate a wide range of cellular processes that are critical for tumorigenesis, including proliferation, survival, and growth [12,14]. Treatment with the flavonoid-based synthetic PI3K inhibitor, LY294002, has been reported to induce antitumorigenic effects, apoptosis, cell growth arrest, blocking tumor cell invasion, and migration in a variety of tumor models [12]. LY294002 was reported to inhibit MMP9 expression and invasion of glioblastoma (C6) cells [39].

NFκB activity is known to be essential for the oncogenic transformation induced by PI3K and AKT [20]. Previous reports have shown a link between AKT and NFκB in that AKT is actively involved in regulating the transcriptional activity of NFκB [13,19,20]. Moreover, blocking NFκB activity was found to be associated with suppression of tumorigenicity [19].

Among mutations in tumor suppressors that are common in lung adenocarcinomas, those of the *TP53* gene occur at high frequency [40] and were reported in ~34% of NSCLC patients [15,34,41,42]. UV exposure of A549 cells led to decreased levels of PI3K p110α and phosphorylated AKT and increased p53 expression [43]. The tumor suppressor, p53, known to negatively regulate transcription of the PI3K gene, was recently shown to suppress EGFR/PI3K/AKT signaling by a mechanism involving crosstalk with AKT mediated via feedback loops to determine the fate of NSCLC cells [41]. In addition and due in part to p53 downregulation, AKT was found to confer resistance in NSCLC [41].

MMPs are known to participate in degradation of extracellular matrix components resulting in tumor progression [22]. Overexpression of MMP2 and MMP9 has been linked to the progression of various types of cancer including lung cancer and correlates with cell invasion, metastasis, and poor prognosis [24,25,26]. AKT has been shown to activate NFκB, known to regulate the transcription of MMP2/9 [13,21]. Extracellularly, proBDNF is known to be cleaved by proteases such as MMP9 to yield C-terminal mBDNF (Figure 1) [3,8,10]. Based on these reports, we hypothesized that differences in the levels of proBDNF and mBDNF in the media of A549 and H1299 cells might be, in part, due to p53-dependent regulation of PI3K/AKT, NFκB, and MMP2/9.

**Figure 3:**
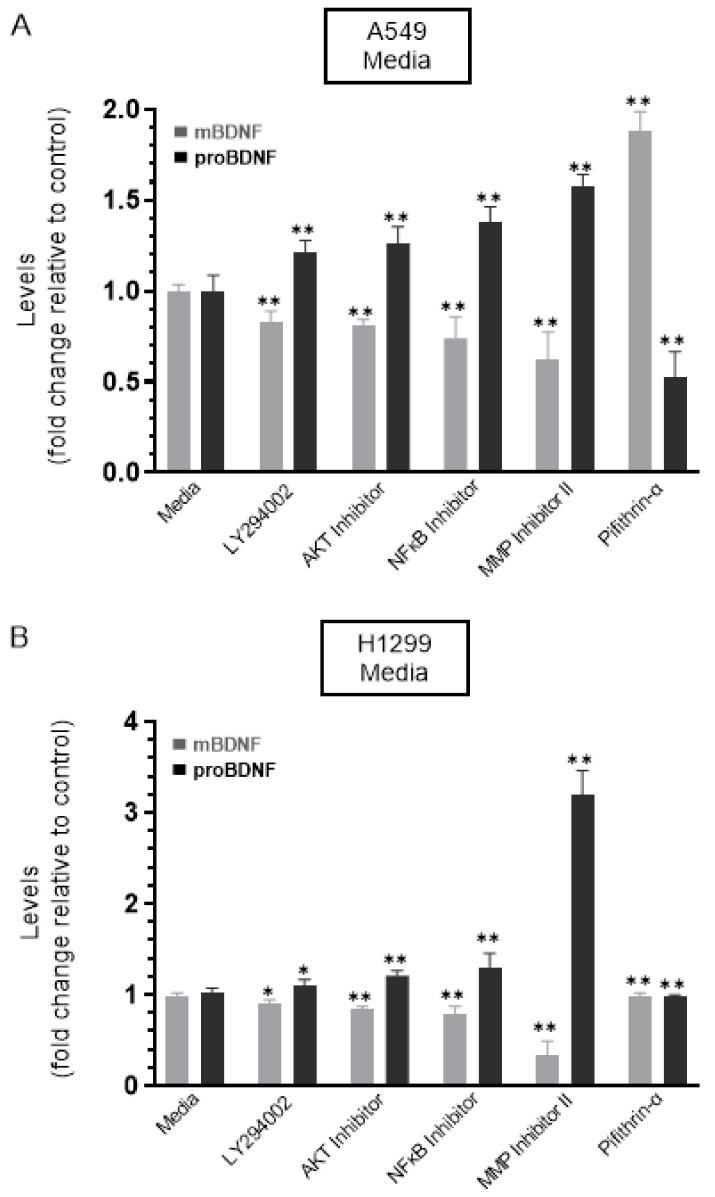
Treatment of cells with the MMP2/9 inhibitor resulted in a comparable decrease and increase in the levels of mBDNF and proBDNF, respectively, in the media of both A549 and H1299 cells, while the opposite was observed upon treatment with the p53 inhibitor, pifithrin-α, in A549 cell media only. Cells (0.2 × 10^5^) were grown in 10% FBS-supplemented media for 24 h. The following day, the cell monolayers were incubated in serum-free media for 24 h, then treated as indicated for 72 h with the inhibitors, as described in the Methods Section. The media was collected, and then the same amount of protein (3 µL of 600 µg/mL total protein) of each sample was used to quantitate mBDNF and proBDNF (Methods) in media from A549 (**A**) and H1299 (**B**) cells. Data from five independent assays, each carried out in triplicate, were quantitated, averaged, normalized, and expressed as fold change relative to control media in the absence of added inhibitors using the GraphPad 9.0.2 software. The graphs summarize the results expressed as means ± SD (*n* = 5). Asterisks (*) indicate a statistically significant difference from the corresponding cell line control, * *p* < 0.05, ** *p* < 0.01, Mann–Whitney test.

Cells were grown in 10% FBS-supplemented media for 24 h. The following day, the cell monolayers were incubated in serum-free media for 24 h and then treated as indicated for 72 h with the inhibitors, as described in the Methods Section and as we recently reported [44]. The media were collected, and then the same amount of protein of each sample was used to quantitate mBDNF and proBDNF (Methods). In the media of both cell lines, there was an increase in the levels of proBDNF and a decrease in the levels of mBDNF upon cell treatment with the PI3K inhibitor, AKT inhibitor, and NFκB inhibitor (Figure 3) suggesting that these proteins are likely involved in the mechanism regulating the ratio of proBDNF/mBDNF in the media of A549 and H1299 cells. Of all the inhibitors examined, treatment with the MMP2/9 inhibitor resulted in the largest increase in proBDNF levels and corresponded with the greatest decrease in the levels of mBDNF in the media of both A549 and H1299 cells (Figure 3). Moreover, a larger fold increase in proBDNF levels, ~3.2-fold, was observed in the media of H1299 cells treated with the MMP2/9 inhibitor as compared to that observed in the media of A549 cells, ~1.6-fold. These results might be indicative of the presence of more mBDNF in H1299 cell media that becomes less pronounced by MMP2/9 inhibition.

No change in the levels of either proBDNF or mBDNF was found upon treatment of H1299 cells with the p53 inhibitor, pifithrin-α, which is not surprising since these cells are p53-negative (Figure 3B). Treatment of A549 cells with pifithrin-α, however, resulted in ~1.85-fold increase in mBDNF and decrease in proBDNF levels compared to control (Figure 3A). These findings suggest that p53 function can account, in part, for the differences in the proBDNF and mBDNF levels in the media of A549 and H1299 cells.

### 2.3. Treatment with the p53 Inhibitor, Pifithrin-α, Resulted in Higher Levels of MMP2 and MMP9 in the Conditioned Media of A549 Cells but Not in H1299 Cell Media

Treatment with the PI3K inhibitor, LY294002, was shown earlier to have apoptotic and antitumorigenic effects leading to attenuation of cell growth, blocking tumor cell invasion and migration in a number of tumor models [12]. Previous reports also showed that MMP9 expression and invasion of glioblastoma (C6) cells were blocked by LY294002 [39].

The tumor suppressor gene, TP53, is widely reported to exhibit inhibitory effects on cell growth, promoting apoptosis when overexpressed in a wide range of tumor cells [15,16,34,42,45]. Lung carcinoma invasion increased upon inactivation of p53 in vitro suggesting a function for p53 in restraining cell invasion and metastasis [46]. Wild-type p53 was shown by comprehensive proteomic analyses to alter the secretome, regulating a variety of secreted proteins including the MMPs [45]. Among the mechanisms that underlie p53-mediated tumor suppression, one was shown to include downregulating cell invasion and decreased expression of MMP2 and secreted levels in human melanoma cell conditioned media, with no modulation of MMP9 secreted levels [46]. Earlier reports found that p53 modulated MMP2 expression [30]. Both p53 and the homeobox transcription factor, HOXA5, were found to cooperate to downregulate tumor cell invasion in NSCLC, in part by inhibiting MMP2 activity [47]. However, while coexpression of p53 and HOXA5 blocked invasion and decreased the levels of MMP2 expression in H1299 cells, no effects were found on the expression of MMP9 [47].

**Figure 4:**
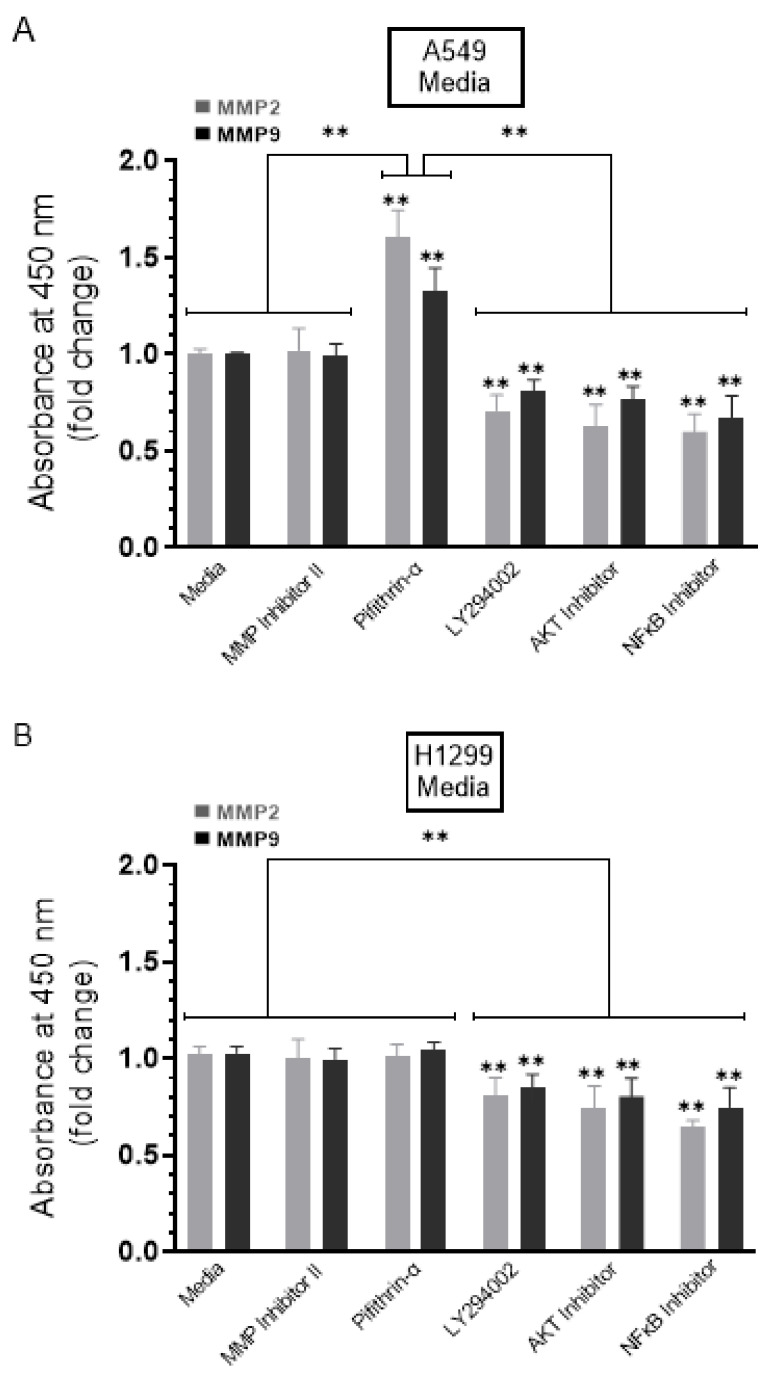
Higher levels of MMP2 and MMP9 were found in the conditioned media of A549 cells upon treatment with the p53 inhibitor, pifithrin-α. Cells (0.2 × 10^5^) were grown in 10% FBS-supplemented media for 24 h. The following day, the cell monolayers were incubated in serum-free media for 24 h and then treated as indicated for 72 h with the inhibitors as described in the Methods Section. The media was collected then the same amount of protein (3 µL of 600 µg/mL total protein) of each sample was used to quantitate MMPs (Methods) in media from A549 (**A**) and H1299 (**B**) cells. Data from five independent assays, each carried out in triplicate, were quantitated, averaged, normalized, and expressed as fold change relative to cells not treated with inhibitors (Media) using the GraphPad 9.0.2 software. The graphs summarize the results expressed as means ± SD (*n* = 5). Asterisks (*) indicate a statistically significant difference from the samples (Media) without inhibitor treatment for each cell line, Mann–Whitney test. Statistical differences between different groups were analyzed by an ordinary one-way analysis of variance (ANOVA) followed by Tukey’s post hoc multiple comparison test. ** *p* < 0.01.

We, therefore, evaluated the levels of MMP2 and MMP9 in the conditioned media of A549 and H1299 cells in the absence or presence of treatment using the different inhibitors (Figure 4) (Methods). No change in the levels of MMP2/9 was detected in the presence of the MMP2/9 inhibitor in the media of either cell line (Figure 4). Increased levels of both MMP2 (~1.55-fold increase) and MMP9 (~1.25-fold increase) were detected in the media upon treatment of A549 cells (Figure 4A) with the p53 inhibitor, pifithrin-α, while there was no effect with this treatment on the levels of MMP2/9 in the media of the p53-null H1299 cells (Figure 4B). These results suggest that p53 functions to decrease MMP2/9 levels in the media of A549 cells. The levels of both MMP2 and MMP9 decreased in the media of both cell lines upon treatment of the cells with the PI3K inhibitor, AKT inhibitor, or NFκB inhibitor (Figure 4), suggesting that these proteins play a role in the mechanism regulating the levels of MMP2 and MMP9 in the media of A549 and H1299 cells.

### 2.4. Treatment of A549 Cells with the p53 Inhibitor, Pifithrin-α, Resulted in Upregulation of PI3K and AKT Activities and the Phospho/Total NFκB Ratio

The PI3K/AKT pathway is known to act via a variety of substrates to promote cancer cell invasion and metastasis [12]. The PI3K/AKT and p53 pathways have been shown to intersect at a number of points and through different mechanisms that include modulation of the PI3K/AKT signaling pathway by the ability of p53 to regulate expression of the PTEN tumor suppressor [48]. Previous findings showed that UV exposure of A549 cells led to increased p53 expression and reduced levels of the PI3K p110α subunit and phosphorylated AKT [43].

In both the N-terminal Rel homology domain and the C-terminal transactivation domain, a number of phosphorylation sites have been mapped on the p65 subunit of NFκB [49]. Phosphorylation of S536 on the p65 subunit of NFκB in the C-terminal transactivation domain is known to be linked with translocation of NFκB subunits and increased NFκB transactivation, while decreased S536 phosphorylation led to inhibition of NFκB activity and cell survival [50].

**Figure 5:**
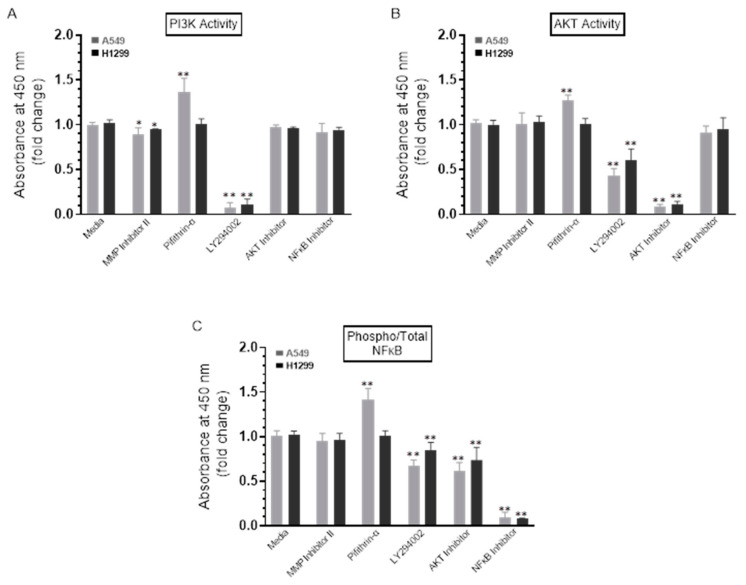
The activity of either PI3K or AKT and the phospho/Total NFκB ratio are upregulated upon treatment of A549 cells with the p53 inhibitor, pifithrin-α. Cells (0.2 × 10^5^) were grown in 10% FBS-supplemented media for 24 h. The following day, the cell monolayers were incubated in serum-free media for 24 h and then treated as indicated for 72 h with the inhibitors. The PI3K activity (**A**) was assayed by the total in-cell ELISA Kit, and the AKT (**B**) and NFκB (**C**) activities were measured on the same amount of protein (3 µL of 600 µg/mL total protein) of the cell lysate as described in the Methods Section. Data from five independent assays, each carried out in triplicate, were quantitated, averaged, normalized, and expressed as fold change relative to cells not treated with inhibitors (Media) using the GraphPad 9.0.2 software. The graphs summarize the results expressed as means ± SD (*n* = 5). Asterisks (*) indicate a statistically significant difference from the corresponding samples without inhibitor treatment (Media) for each cell line, Mann–Whitney test, while the absence of asterisks indicates no significance. * *p* < 0.05, ** *p* < 0.01.

To test the effect of the inhibitors on the activity of PI3K, AKT, and NFκB, cells were treated with the inhibitors as indicated, and then the PI3K, AKT, and NFκB assays were performed as described in the Methods Section. A549 cell treatment with the p53 inhibitor, pifithrin-α, resulted in a ~1.30-fold increase in the activities of PI3K and AKT (Figure 5A,B). No effects were found when H1299 cells were treated with pifithrin-α (Figure 5A,B), which is not surprising since they are p53-negative [34]. These results might indicate downregulation of PI3K and AKT activities by p53 in A549 cells.

No apparent effects were detected upon addition of the MMP2/9 inhibitor on the ratio of phospho/total NFκB (Figure 5C) in either A549 or H1299 cells. Treatment of A549 cells, however, with LY294002 resulted in ~1.40-fold decrease in phosphorylation, while a ~1.55-fold decrease was observed in phosphorylation upon treatment of A549 cells with the AKT inhibitor (Figure 5C). While similar trends were observed, more modest effects were found when H1299 cells were treated with LY294002 and the AKT inhibitor resulting in a decrease of ~1.15-fold and ~1.30-fold, respectively, in NFκB phosphorylation (Figure 5C). Expectedly, no effects were observed when treating the p53-null H1299 cells with the p53 inhibitor, pifithrin-α; however, A549 cell treatment with this inhibitor increased NFκB phosphorylation by ~1.45-fold (Figure 5C). In accordance with previous findings showing that NFκB and p53 have opposing effects in cancer cells with antagonistic signaling, crossregulating each other’s activity and suppressing each other’s ability to enhance gene expression, [18] our observation that NFκB phosphorylation is increased by inhibiting p53 (Figure 5C) suggests that p53 acts as an antagonist of NFκB phosphorylation in A549 cells.

### 2.5. MMP9-siRNA Transfections Resulted in Decreased Levels of mBDNF and a Corresponding Increase in ProBDNF in the Media of A549 and H1299 Cells, while the Converse Was Observed upon Transfection of A549 Cells with p53 siRNA

In examining the levels of amyloid beta, we have previously found that treatment with the p53 inhibitor, pifithrin-α, led to higher levels of MMP2 and MMP9 in A549 cell-conditioned media, while no effects were observed on either MMP2 or MMP9 levels with this treatment in the p53-null H1299 cell media, suggesting that p53 functions to decrease MMP2/9 levels in the media of A549 cells [44]. These results are consistent with our findings in this study (Figure 4).

**Figure 6:**
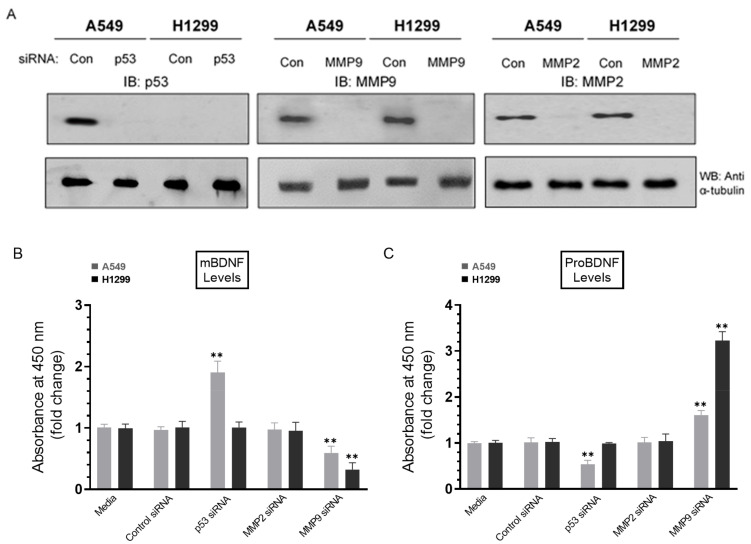
Treatment of A549 cells with p53 siRNA resulted in increased levels of mBDNF while treatment with MMP9 siRNA decreased those levels in the media of both cell lines and corresponded with increased proBDNF. Cells (0.2 × 10^5^) were grown in 10% FBS-supplemented media for 24 h. The following day, the cell monolayers were incubated in serum-free media for 24 h and then treated for 72 h with the indicated siRNAs as described in the Methods Section. The same concentration of total protein (15 µL of 600 µg/mL) of the cell lysates (**A**) was used for Western blotting using the indicated antibodies. As a loading control, anti α-tubulin antibodies were used. The same amount of protein (3 µL of 600 µg/mL total protein) of the media was used to quantitate the levels of mBDNF (**B**) and proBDNF (**C**) (Methods). Data from five independent assays, each carried out in triplicate, were quantitated, averaged, normalized, and expressed as fold change relative to cells transfected with control siRNA using the GraphPad 9.0.2 software. The graphs summarize the results expressed as means ± SD (*n* = 5). Asterisks (*) indicate a statistically significant difference from the corresponding samples transfected with control siRNA, ** *p* < 0.01 of each cell line. Absence of asterisks indicates no significance, Mann–Whitney test.

When compared with other inhibitors (Figure 3), we found that inhibition of MMP2/9 led to the largest increase in proBDNF and decrease in mBDNF levels in the media of A549 and H1299 cells. Moreover, p53 appears to be an important regulator since inhibiting its function with pifithrin-α resulted in increased and decreased levels of mBDNF and proBDNF, respectively, in A549 cell media (Figure 3). Therefore, using siRNA targeted against either MMP, we next examined the relative contributions of MMP2 or MMP9 on the levels of proBDNF and mBDNF (Methods) (Figure 6). In addition, to further verify the involvement of p53, we tested the effects of treating the cells with p53 siRNA on proBDNF and mBDNF levels in the conditioned media (Figure 6).

Cells were grown in 10% FBS-supplemented media for 24 h and then incubated in serum-free media for 24 h, followed by treatment for 72 h with the indicated siRNAs as described in the Methods Section (Figure 6). Treatment of A549 cells with p53 siRNA led to ~2.0-fold increase in mBDNF levels (Figure 6B) and ~1.85-fold decrease in the levels of proBDNF (Figure 6C) relative to cells transfected with control siRNA. This decrease was comparable to that observed upon inhibition of p53 using pifithrin-α (Figure 3A), suggesting that the function of p53 is important for regulating the levels of proBDNF and mBDNF in A549 cell media. No effects were detected on the levels of either proBDNF or mBDNF in H1299 cells transfected with p53 siRNA (Figure 6), giving results consistent with the lack of p53 in H1299 cells. Compared to p53 transfections, opposite effects were observed on the levels of mBDNF and proBDNF upon cell treatment with MMP9 siRNA (Figure 6B,C). Transfection of A549 cells with MMP9 siRNA resulted in ~1.65-fold change in the levels of proBDNF and mBDNF, while a greater fold change, ~3.15-fold, was observed upon transfection of H1299 cells with MMP9 siRNA (Figure 6). No effects were observed upon transfection of either cell line with MMP2 siRNA (Figure 6). These results clearly highlight the importance of MMP9 in regulating the proBDNF and mBDNF levels in lung cancer cell media.

### 2.6. Incubation with Exogenously Added MMP9, but Not MMP2, Resulted in Higher Levels of mBDNF and a Concomitant Decrease in the Levels of ProBDNF in the Media of A549 Cells, Effects That Were Relatively Minimal in H1299 Cell Media

Our results show that transfection using MMP9 siRNA led to decreased levels of mBDNF and a corresponding increase in the levels of proBDNF in the media of A549 and H1299 cells (Figure 6) with no effects observed upon transfection of either cell line with MMP2 siRNA (Figure 6). These results suggest a functional role for MMP9 in regulating the proBDNF and mBDNF levels in lung cancer cell media. Previous reports have shown that MMP2 is unable to convert proBDNF to mBDNF, despite comparable enzymatic characteristics of MMP2 to those of MMP9 [51,52].

**Figure 7:**
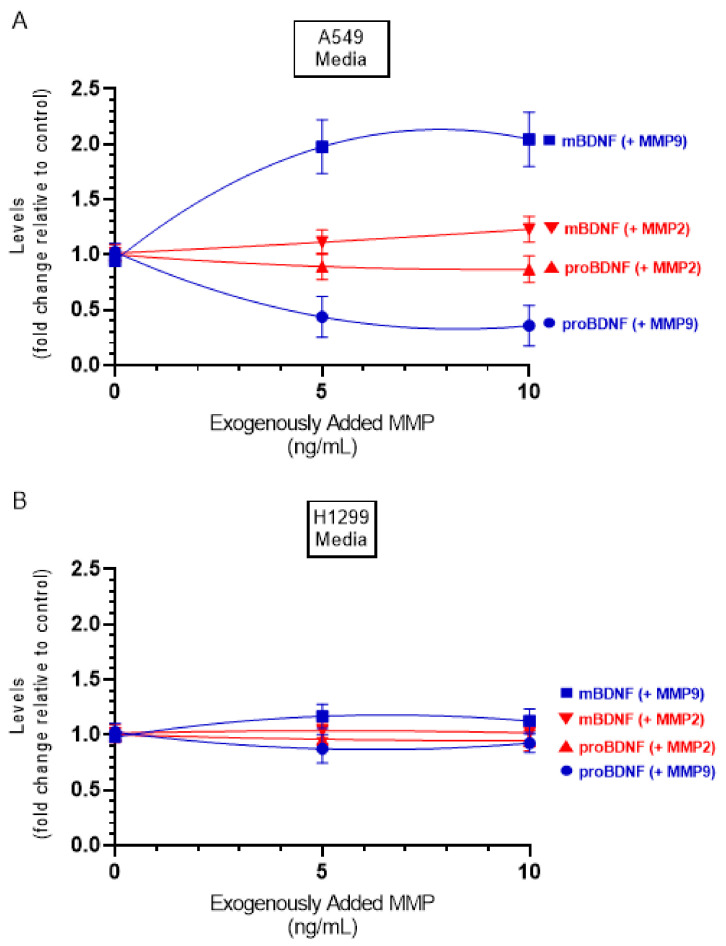
Addition of active MMP9 enzyme increased the mBDNF/proBDNF ratio in A549 cell media, while minimal effects were observed using active MMP2 enzyme. Cells (0.2 × 10^5^) were grown in 10% FBS-supplemented media for 24 h in 25 cm^2^ flasks then serum starved for 48 h. Active MMP2 or MMP9 enzymes were then added at the indicated concentrations to the media, and the cells were allowed to incubate at 37 °C overnight. The same concentration of total protein (3 µL of 600 µg/mL) of the media was then used to quantitate mBDNF and proBDNF (Methods) in media from A549 (**A**) and H1299 (**B**) cells. Data from three independent assays, each carried out in triplicate, were quantitated, averaged, normalized, and expressed as fold change relative to the corresponding control without added MMP. Data were calculated and fit using the GraphPad 9.0.2 software with a nonlinear regression curve. The data represent the mean ± SD of three separate experiments, each performed in triplicate.

In this study, the levels of MMP2/9 were quantitated as described in the Methods Section and found to be ~3 ng/mL and ~5 ng/mL in the media of A549 and H1299 cells, respectively. To examine whether increasing concentrations of exogenously added MMP2 or MMP9 alters the ratio of mBDNF/proBDNF, active MMP enzymes were added at the indicated concentrations (Figure 7), and then the levels of mBDNF and proBDNF were quantitated (Methods). Exogenously added MMP9 enzyme at a concentration of 5 ng/mL to A549 cell media, resulted in ~2.00-fold increase in the levels of mBDNF with a concomitant decrease in the levels of proBDNF. No more apparent increase was found upon addition of 10 ng/mL protein possibly indicating efficient proteolysis of proBDNF to form mBDNF. Incubation with exogenously added active MMP2 enzyme had a relatively smaller effect possibly suggesting the lack of efficient proBDNF to mBDNF conversion by MMP2. While the trends were similar using H1299 cell media, the effects were almost negligible compared to those found using A549 cells, an observation that might be due to the relatively lower levels of proBDNF in H1299 cell media (Figure 2).

### 2.7. MMP9 siRNA Treatment of Either A549 or H1299 Cells Decreased Cell Viability and Increased Apoptosis, an Effect Diminished upon the Same Treatment with ProBDNF Immunodepleted Media

**Figure 8:**
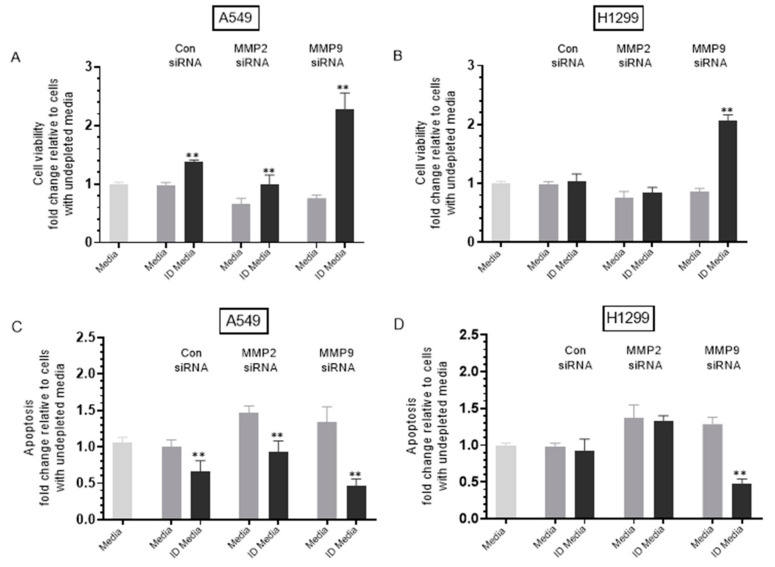
Treatment of either A549 or H1299 cells with MMP9 siRNA decreased cell viability and increased apoptosis, an effect reduced upon treatment with proBDNF immunodepleted (ID) media. ProBDNF immunodepleted media (ID) was prepared by growing cells (0.2 × 10^5^) in 10% FBS-supplemented media for 24 h. The cells were then incubated in serum-free media overnight then with the indicated treatments for 72 h. The media were then collected and depleted from proBDNF using anti-proBDNF specific antibodies (Methods). Viability (**A**,**B**) and apoptosis (**C**,**D**) of A549 and H1299 cells were assessed as described in the Methods Section. In brief, cells were seeded in 96-well plates at 0.2 × 10^5^ cells per well in 10% FBS-supplemented media. The next day, the cell monolayers were incubated in serum-free media for 12 h, then treated with 300 μL of the control and proBDNF ID media (0.5 μg/μL) for 72 h with the media containing the specific components in the various treatments replaced every 12 h. Data were processed using the GraphPad 9.0.2 software. The graphs summarize the results expressed as means ± SD (*n* = 3) of three separate experiments, each performed in triplicate. Asterisks (*) indicate a statistically significant difference between each treatment relative to nondepleted samples, ** *p* < 0.01. Absence of asterisks indicates no significance, Mann–Whitney test.

Our results (Figure 6) show that MMP9 siRNA transfection of either A549 or H1299 cells resulted in increased proBDNF levels and that incubation with exogenously added MMP9, but not MMP2, resulted in higher levels of mBDNF which corresponded to decreased proBDNF levels in the media of A549 cells (Figure 7). We next asked whether these increased levels of proBDNF upon transfection with MMP9 siRNA had an effect on cell viability or apoptosis (Figure 8). To address this question, media was first collected from different cell treatments, then immunodepleted (ID) of proBDNF (Methods). Next, viability and apoptosis of A549 and H1299 cells were assessed as described in the Methods Section and in Figure 8 legend.

Immunodepletion of proBDNF from media of A549 cells transfected with control siRNA resulted in ~1.45-fold increase in viability and ~1.52-fold decrease in apoptosis (Figure 8A,C), suggesting that proBDNF has cytotoxic functions in the media of this cell line. No effects were observed under these conditions (Figure 8B,D) using H1299 cells, possibly suggesting the lack of sufficient concentrations of proBDNF in the media of this cell line.

Treatment of A549 cells with MMP2 siRNA media decreased cell viability and increased apoptosis by ~1.45-fold (Figure 8A,C). Immunodepletion of proBDNF from media of A549 cells treated with MMP2 siRNA resulted in ~1.52-fold increase in viability (Figure 8A) and ~1.58-fold decrease in apoptosis (Figure 8C) compared to undepleted media. The effects observed with proBDNF immunodepletions of MMP2 siRNA treatment were close to those obtained using control siRNA. This observation might be explained by the lack of effect on proBDNF levels upon transfection with MMP2 siRNA (Figure 6C) or as compared to exogenously added MMP9, the relatively inefficient conversion of proBDNF to mBDNF upon addition of exogenous MMP2 enzyme (Figure 7). Transfection of H1299 cells with MMP2 siRNA resulted in ~1.35-fold decrease in viability and increase in apoptosis (Figure 8B,D). However, no change was observed upon proBDNF immunodepletion, further supporting the hypothesis that MMP2 does not significantly modulate the effects of proBDNF.

Treatment of A549 cells with media from MMP9 siRNA transfection resulted in ~1.30-fold decrease in A549 cell viability, and increase in apoptosis (Figure 8A,C). Immunodepletion of proBDNF from MMP9 siRNA transfected A549 cell media resulted in ~3.00-fold increase in cell viability and ~2.92-fold decrease in apoptosis (Figure 8A,C). Transfection of H1299 cells with MMP9 siRNA resulted in ~1.15-fold decrease in viability and ~1.20-fold increase in apoptosis (Figure 8B,D). Immunodepletion of proBDNF from the media of H1299 cells transfected with MMP9 siRNA resulted in ~2.40-fold increase in cell viability and ~2.70-fold decrease in apoptosis (Figure 8B,D) compared to undepleted media.

**Figure 9:**
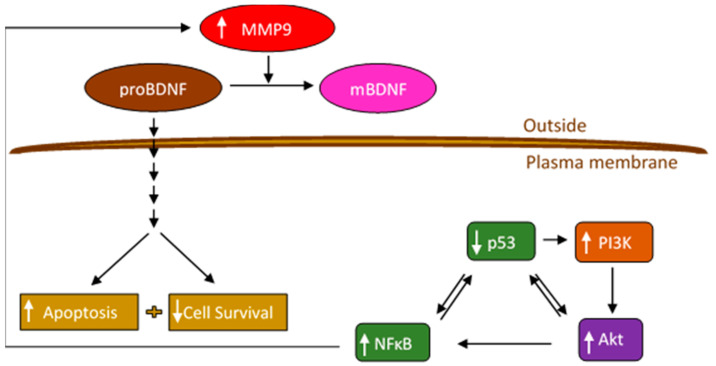
Representation of the main hypothesis and findings of this study.

These results suggest that treatment with MMP9 siRNA leads to an increase in proBDNF cytotoxicity in both cell lines compared to that found with MMP2 siRNA treatment. This observation might also suggest that, of the two MMPs, MMP9 is the predominant regulator of proBDNF cytotoxic functions in the media of these cell lines. Moreover, these results might correlate with the findings (Figure 6) showing the relatively higher levels of proBDNF in the media upon treatment with MMP9 siRNA as compared to MMP2 siRNA treatment in both cell lines. While the enzymatic characteristics of MMP2 are comparable to those of MMP9, MMP2 was reported earlier to lack the ability to convert proBDNF to mBDNF [51,52]. Taken together, our results suggest that cell treatment with MMP9 siRNA leads to increased levels of proBDNF resulting in cytotoxicity (Figure 9).

## 3. Discussion

Accumulating evidence now links cancer and neurodegeneration disease mechanisms, and several emerging overlapping molecular pathways have been identified in these mechanisms [53,54,55]. An inverse correlation has been observed between the likelihood of developing a cancer and a neurodegenerative disorder, with reduced incidence for most cancers for those affected by a neurodegenerative disorder [53,54,55].

Earlier, we showed that immunodepletion of the neuroprotective peptide, humanin, from the media of A549 and H1299 cells increased the relative abundance of oligomer vs. total levels of amyloid beta, a peptide involved in the Alzheimer disease process, and was correlated with diminished cell viability and increased apoptosis [56]. We then found that ATP, thought to reduce misfolding of amyloid beta, strengthened interactions between amyloid beta and humanin [57]. More recently, we showed that higher intact amyloid beta 40/42 levels are present in the media of A549 (p53 wild-type) than in H1299 (p53-null) lung cancer cell media [44].

To further examine the function of other molecular players implicated in overlapping mechanisms that converge at the interface of neurodegeneration and cancer, we chose to focus on BDNF in lung cancer cells in this study. The levels and activities of BDNF have been described in a number of neurodegenerative disorders with the protein widely recognized for its role in the survival of neurons and synapses [3,58]. There is increasing recognition, however, of the emerging roles of neurotrophins in a number of cancers [3,4,5]. Secretion of BDNF has been reported in tumor cells, promoting cell growth and survival of a variety of cancers including lung [7,8]. As is the case for all neurotrophins, BDNF is first synthesized as a proneurotrophin precursor form, proBDNF [10]. Opposing functional roles have been attributed to secreted BDNF and proBDNF on cell proliferation and apoptosis [6,8]. In view of the differences we found in the intact levels of amyloid beta 40/42 in the conditioned media of lung cancer cell lines, [44] we asked whether there are also differences in the levels of proBDNF and mBDNF in the media of these cells. Our results show that proBDNF levels are higher in A549 (p53-positive) cell media than in the media of H1299 (p53-null) cells (Figure 2). Two bands were detected in the media of A549 cells by Western blotting using anti-mBDNF antibodies, while one band was observed in the media of H1299 cells (Figure 2A). These antibodies are expected to bind an epitope in the mBDNF sequence (Figure 1) and, therefore, are expected to also bind that sequence in the proBDNF peptide (Amino acids 19-247, Figure 1). To more precisely quantitate the levels of mBDNF, quantitation of mBDNF (Biosensis, Methods) was carried out using an anti-mBDNF capture antibody and a biotinylated anti-mBDNF detection antibody. The levels of mBDNF were found to be higher in H1299 cell media compared to the media of A549 cells (Figure 2C). Conversely, using an anti-proBDNF capture antibody and a biotinylated anti-mBDNF detection antibody (Biosensis, Methods) to quantitate proBDNF, we found higher levels of proBDNF in A549 cell media than in the media of H1299 cells (Figure 2C). The differences in these levels might be indicative of a plausible mechanism through which there is increased proteolytic cleavage of proBDNF to mBDNF in H1299 cell media. Using inhibitors in an effort to identify signaling molecules that play a role in regulating the levels of proBDNF and mBDNF, we found increased levels of proBDNF and a corresponding decrease in mBDNF levels in the media of both cell lines upon cell treatment with the PI3K inhibitor, AKT inhibitor, and NFκB inhibitor (Figure 3) likely indicating the involvement of these proteins in the mechanism regulating the ratio of proBDNF/mBDNF in the media of A549 and H1299 cells. The largest difference in the levels of proBDNF and mBDNF in the media of both A549 and H1299 cells (Figure 3) was observed by treatment with the MMP2/9 inhibitor. A greater fold increase in proBDNF levels, ~3.2-fold, was observed in H1299 cell media treated with the MMP2/9 inhibitor as compared to that detected in the media of A549 cells, ~1.6-fold, possibly suggesting the existence of higher levels of mBDNF in H1299 cell media that become less abundant by MMP2/9 inhibition.

Not surprisingly, treatment of H1299 cells with the p53 inhibitor, pifithrin-α, did not result in changes in the levels of either proBDNF or mBDNF, since they are p53-negative (Figure 3B), while the same treatment of A549 cells resulted in ~1.85-fold increase in mBDNF and decrease in proBDNF levels compared to control (Figure 3A). These results suggest that differences in levels of proBDNF and mBDNF in the media of A549 and H1299 cells might be accounted for, in part, by p53 function. The tumor suppressor gene, *TP53*, has been shown through numerous reports to exert apoptotic functions and inhibitory effects on cell growth when overexpressed in a wide range of tumor cells [15,16,34,42,45]. Lung carcinoma invasion was increased in vitro upon inactivation of p53 suggesting that p53 executes its antineoplastic functions by regulation of cell invasion [46]. Comprehensive proteomic analyses revealed that wild-type p53 regulates the secretome, controlling a wide variety of secreted proteins including MMPs [45]. Treatment with the p53 inhibitor, pifithrin-α, resulted in increased levels of MMP2 and MMP9 in A549 cell conditioned media but not in the media of the p53-null H1299 cells (Figure 4), suggesting that p53 functions to downregulate MMP2/9 levels in A549 cell media. Inhibition of p53 function with pifithrin-α resulted in an increase in the levels of mBDNF and a decrease in proBDNF levels in A549 cell media (Figure 3). A comparable decrease in the levels of proBDNF and increase in the levels of mBDNF were found in the media of A549 cells transfected with p53 siRNA relative to control (Figure 6) as those observed upon inhibition of p53 using pifithrin-α (Figure 3A), an observation pointing to the importance of p53 function in regulating the levels of proBDNF and mBDNF in A549 cell media. Our results (Figure 3) show that inhibition of MMP2/9 led to the largest increase in proBDNF and decrease in mBDNF levels in the media of A549 and H1299 cells compared to the other inhibitors used. To distinguish the role of MMP2 and MMP9 in regulating these levels, siRNA was used to knockdown expression of the proteins (Figure 6). No effects on proBDNF or mBDNF levels were found upon transfection of either cell line with MMP2 siRNA (Figure 6). While the trends were similar for cells transfected with MMP9 siRNA, a greater fold increase in the levels of proBDNF (~3.15-fold) and corresponding decrease in the levels of mBDNF were found in the media of H1299 cells compared to the more modest fold change in proBDNF and mBDNF levels (~1.65-fold) in the media of A549 cells (Figure 6). Consistent with these observations, incubation with exogenously added MMP9, but not MMP2, led to higher mBDNF levels and a concomitant decrease in the levels of proBDNF in the media of A549 cells (Figure 7A). These effects were relatively minimal in H1299 cell media (Figure 7B), possibly due to the low concentrations of proBDNF present in the media of this cell line compared to those found in the media of A549 cells (Figure 2). Our results clearly highlight the importance of MMP9, of the two MMPs, in regulating the levels of proBDNF and mBDNF in lung cancer cell media and might be, in part, explained by previous reports showing that MMP2 is not able to convert proBDNF to Mbdnf [51,52].

It is generally accepted that mBDNF and proBDNF often function opposite to control cell proliferation and apoptosis [6,8]. Several studies have reported that proneurotrophins trigger cell death, whereas mature neurotrophins increase cell survival [9]. Upon binding of BDNF to its receptor, a series of downstream pathways are stimulated, including the PI3K/AKT pathway, inducing oncogenic effects by promoting cell growth and survival [8]. BDNF was shown to be upregulated in lung cancer cells compared to normal lung cells and critical for lung tumorigenesis [7]. Using C6 glioma cells, proBDNF was shown to increase apoptosis and decrease cell growth, while the ratio of proBDNF/mBDNF was decreased in high-grade glioma, negatively correlating with tumor grade [11]. Since our results (Figure 6) show that transfection of either A549 or H1299 cells with MMP9 siRNA resulted in increased proBDNF levels, we tested whether these increased levels of proBDNF had an effect on cell viability or apoptosis (Figure 8). Immunodepletion of proBDNF from media of A549 cells transfected with control siRNA resulted in increased cell viability and a corresponding decrease in apoptosis (Figure 8A,C), suggesting that proBDNF has cytotoxic functions in the media of A549 cells. No effects were found under these conditions (Figure 8B,D) using H1299 cells, a result that might be explained by the lack of sufficient concentrations of proBDNF in the media of this cell line relative to that of A549 cells (Figure 2). In contrast to the results with the MMP9 transfections, the effects observed with proBDNF immunodepletions of media of A549 or H1299 cells transfected with MMP2 siRNA were comparable to those obtained using control siRNA (Figure 8), a finding that might be due to the lack of effect on proBDNF levels upon transfection with MMP2 siRNA (Figure 6C). Relative to undepleted media, proBDNF immunodepletion from MMP9 siRNA transfected A549 cell media resulted in ~3.00-fold increase in cell viability and a comparable fold decrease in apoptosis (Figure 8A,C), while immunodepletion of proBDNF from the media of H1299 cells transfected with MMP9 siRNA resulted in ~2.40-fold increase in cell viability and a similar fold decrease in apoptosis (Figure 8B,D). These observations might indicate that treatment with MMP9 siRNA leads to an increase in proBDNF levels (Figure 6) and resulting cytotoxicity (Figure 8) in both cell lines.

A link between AKT and NFκB has been previously reported in that AKT regulates the transcriptional activity of NFκB, while high levels of NFκB were found to be essential for oncogenic transformations induced by PI3K and AKT [13,19,20]. Signaling by NFκB and p53 has been reported to engage antagonistic crosstalk and reciprocal negative regulation at multiple levels in cancer cells [18]. NFκB and p53 were both reported to crossregulate each other’s function and inhibit each other’s ability to increase gene expression [18]. NFκB activity was found to shut down p53 function and its responses and be increased in p53-null mice, and p53 loss triggered NFκB activation in a mouse model of KrasG12D-driven lung adenocarcinoma [45,59]. Conversely, restoring p53 in p53-null lung tumors resulted in NFκB inhibition and tumor suppression [45,59].

Treatment of A549 and H1299 cells with the PI3K inhibitor, AKT inhibitor, or NFκB inhibitor led to decreased levels of both MMP2 and MMP9 in the media (Figure 4), suggesting the likely involvement of these proteins in the mechanism regulating the levels of MMP2/9 in the media of these lung cancer cells. We also found higher levels of MMP2 and MMP9 in A549 cell conditioned media (Figure 4) and upregulation of PI3K and AKT activities along with an increased phospho/total NFκB ratio (Figure 5) upon treatment of A549 cells with the p53 inhibitor, pifithrin-α, in this study and in our previously published report [44]. The findings from this study might also lead to the hypothesis that, of the two MMPs, MMP9 is the predominant regulator of proBDNF proteolysis (Figure 6 and Figure 7) and resulting effect on cytotoxicity (Figure 8) and suggest that proteolytic cleavage of proBDNF by MMP9 represents a mechanism (Figure 9) by which the opposing actions of proBDNF and mBDNF may be regulated in the media of lung cancer cell lines.

While results from this study suggest a functional role for p53 in regulating the levels of mBDNF and proBDNF in NSCLC, it is important to note that although A549 cells are p53-positive and H1299 cells are p53-null, the A549 cell line carries a KRASG12S mutation and is of epithelial origin, whereas the H1299 cell line expresses a KRAS WT and is of mesenchymal origin [60,61,62]. The KRAS gene is known to be characterized by single base missense mutations, found predominantly at codons G12, G13, or Q61 [63,64]. Expression of KRASG12C in A549 cells has been previously shown to mediate its oncogenic signaling via the MAPK and PI3K/AKT/mTORC1/p70S6K signaling pathways [60,65] while disruption of the KRASG12S allele blocked AKT and ERK signaling pathways, inhibiting tumor growth and proliferation [65]. Since mutant KRAS has been shown to interact with NFκB-activating kinases promoting cancer cell survival and drug resistance [66], it is important to examine the potential role of mutant KRAS in regulating the levels and/or ratio of proBDNF/mBDNF. Work in our laboratory is currently underway to examine this role using A549 (p53Wt/KRASG12S), H358 (p53Null/KRASG12C), and H1299 (p53Null/KRASWt) cell lines.

## 4. Materials and Methods

### 4.1. Materials

Most of the material used in this study was purchased as we reported earlier [56,67,68,69]. Phosphate-buffered saline (PBS), nitrocellulose membranes, streptavidin-horseradish peroxidase (HRP) conjugate, Ponceau S solution, LY294002 hydrochloride, hydrogen peroxide solution, AKT Inhibitor (Calbiochem, San Diego, CA, USA), pifithrin-α p-Nitro, active human MMP9 (PF024), and active human MMP2 (PF023) were purchased from Sigma-Aldrich, St. Louis, MO, USA. MMP2/MMP9 Inhibitor II was purchased from EMD Millipore (Burlington, MA, USA). Sheep BDNF polyclonal antibody (PA1-18363), donkey antisheep IgG (H+L) secondary antibody (HRP, A16041), rabbit proBDNF polyclonal antibody (PA1-18374), goat antirabbit IgG (H+L) secondary antibody (HRP, 31466), mouse IgG isotype control, (mIgG), α-tubulin monoclonal antibody (DM1A), 3,3′,5,5′-tetramethylbenzidine (TMB), and lipofectamine 2000 transfection reagent were from ThermoFisher (Waltham, MA, USA). Rabbit antigoat IgG (HRP) (ab6741) and donkey antimouse IgG (HRP) (ab205724) were purchased from Abcam (Cambridge, MA, USA). Goat antirabbit IgG-HRP (sc-2004), NFκB inhibitor (CAS 213546-53-3), MMP2 siRNA (sc-29398), MMP2 antibody (sc-13594), MMP9 siRNA (sc-29400), MMP9 antibody (sc-393859), and m-IgGκ BP-HRP were from Santa Cruz Biotechnology (Dallas, TX, USA). The caspase 3 (cleaved) colorimetric In-Cell ELISA Kit (62218), the BCA protein assay kit, and the super signal west pico luminol (chemiluminescence) reagent were from Pierce (Waltham, MA, USA). SignalSilence p53 siRNA I (6231), SignalSilence Control siRNA (Unconjugated, 6568), and p53 antibody (9282) were purchased from Cell Signaling Technology (Danvers, MA, USA).

### 4.2. Cell Culture

Human NSCLC cell lines, A549 (ATCC CCL-185) and H1299 (ATCC CRL-5803), were purchased from the American Type Culture Collection (ATCC, Manassas, VA, USA). Cells were seeded as we reported earlier [44,56,57,67,68,69] in 5 mL HyClone Dulbecco’s modified Eagle’s media/nutrient mixture F-12 (DMEM/F12) (GE Healthcare Life Sciences, Pittsburgh, PA, USA), supplemented with 10% Fetalgro bovine growth serum (FBS, RMBIO, Missoula, MT, USA), 50 U/mL penicillin, and 50 U/mL streptomycin (Invitrogen Life Technologies, Carlsbad, CA, USA) in 25 cm^2^ tissue culture flasks, and allowed to grow overnight in an incubator at 37 °C, 95% humidity, and 5% CO_2_. The cells were counted after trypan blue staining, with a hemocytometer.

When inhibitors were used, cells were treated with inhibitors targeted against PI3K (LY294002, 14.5 μM), AKT (AKT inhibitor, 1.75 μM), p53 (pifithrin-α, 10 μM), NFκB (NFκB inhibitor, 18 μM), and MMP2/9 (MMP inhibitor II, 5 μM), as indicated.

### 4.3. MMP2/9 Level Measurement

The levels of MMP2/9 in the cell culture media were measured as we previously reported [44] using the Invitrogen human MMP2/9 solid-phase sandwich ELISA kits. In brief, the assay uses a matched antibody pair to measure the amount of the target bound. Samples are added to wells precoated with a target-specific (capture) antibody. The second (detector) antibody is then added, and the signal, proportional to the concentration of the target, is detected after addition of a substrate solution.

### 4.4. PI3K Assay

Activated phosphorylated-PI3K p85 + total PI3K p85 in-cell ELISA kit (Abcam, Cambridge, UK) was used according to the recommendations by the manufacturer as we recently reported [44]. In brief, cells were cultured in 96-well plates then treated as indicated. Following treatment, the cells were fixed, and the wells were then incubated with a primary antibody targeting either total PI3K p85 (recognizes the total level of PI3K p85 proteins regardless of the phosphorylation state) or phosphorylated-PI3K p85 (recognizes p85 PI3K alpha/gamma phospho-tyrosine 467/199). Secondary HRP-conjugated antibodies were then added, and the signal detected after addition of the developing solution. Crystal Violet solution was then added to determine the relative number of cells in each well. Signals for phospho-PI3K and total-PI3K were normalized to cell number, and then the ratio of phospho-PI3K to total-PI3K for each treatment was determined and plotted.

### 4.5. AKT Assay

The AKT kinase activity assay kit (Abcam) was used to quantitate the activity of AKT according to the manufacturer’s instructions as we reported recently [44]. In brief, the assay is based on a solid-phase ELISA. A specific synthetic peptide is used as a substrate for AKT along with a polyclonal antibody that binds the phosphorylated substrate.

### 4.6. NFκB Assay

The NFκB p65 (Phospho/Total) InstantOne sandwich ELISA kit (Invitrogen) was used as we reported previously [44] according to the manufacturer’s recommendations. Signals for phospho (Ser536) and total NFκB were normalized to cell number, and then the ratio of phospho (Ser536) to total NFκB for each treatment was determined and plotted.

### 4.7. MTT Assay

The MTT reduction assay (Sigma-Aldrich, St. Louis, MO, USA), used to measure cell viability, was carried out as we reported earlier [67,69,70]. Cells were seeded in 96-well plates as indicated in 200 μL 10% FBS-supplemented media per well and maintained overnight at 95% humidity and 5% CO_2_. After an overnight incubation, the media were replaced with 200 μL serum-free media, and the cells were further incubated, without or with different treatments, for 24, 48, or 72 h. The final concentration of DMSO in each well, never exceeded 0.1%. The cells were then incubated for 4 h with MTT (0.5 mg/mL) in the dark. The media was carefully removed, and DMSO (100 μL) was added to dissolve the formazan crystals. The absorbance was measured at 570 nm in a plate reader. All absorbance measurements were in the linear range. Untreated cells or wells containing only DMSO and media were used as a positive and negative control, respectively. Statistical analysis was conducted using GraphPad Prism version 9.0.2 for Windows. Significant values were considered at *p* < 0.05 and more significant values at *p* < 0.01, compared with the control.

### 4.8. Apoptosis Assay

For the caspase 3 (cleaved) colorimetric assay, activated (cleaved) caspase 3 and tubulin were simultaneously measured in triplicate in whole cells by a colorimetric in cell ELISA assay (ThermoFisher, Waltham, MA, USA) using 96-well microplates as we previously described [70,71]. In brief, cells were plated per well and incubated overnight at 37 °C in 5% CO_2_. Cells were treated as indicated then subsequently fixed with 4% formaldehyde, permeabilized according to the manufacturer’s instructions and incubated with primary antibodies overnight at 4 °C. HRP conjugates were added next and incubated at RT for 30 min. Subsequent to washing the plates, the TMB substrate was added to each well and incubated in the dark at RT. The reaction was typically stopped after 15 min with a TMB stop solution when the blue color became apparent. The absorbance was then measured at 450 nm within 30 min after the reaction was stopped. Control cells were treated with a 0.1% DMSO vehicle control and contained all the reagents except the primary antibodies. The average of all replicate nonspecific background signal controls from each condition was subtracted then the average absorbance at 450 nm for each condition was calculated.

### 4.9. Quantitation of proBDNF and mBDNF

Quantitation of proBDNF was carried out using the rapid sandwich ELISA kit (Biosensis, BEK-2217-1P). The kit consists of a precoated polyclonal anti-proBDNF capture antibody that binds to epitopes within the prodomain of proBDNF and a biotinylated anti-mBDNF detection antibody. The concentration of proBDNF in the media was detected after addition of HRP-conjugated streptavidin and the substrate, TMB, according to the manufacturer’s protocol. Quantitation of mBDNF was carried out using the mBDNF rapid sandwich ELISA kit (Biosensis, BEK-2211-1P), which shows minimal crossreactivity with proBDNF, according to the instructions provided by the manufacturer. The kit consists of a precoated mouse monoclonal anti-mBDNF capture antibody and a biotinylated anti-mBDNF detection antibody. The concentration of mBDNF in the media was calculated after addition of HRP-conjugated streptavidin and the TMB substrate.

### 4.10. Immunodepletion

Conditioned media was immunodepleted (ID) according to methods previously described [72] and our recently published reports [44,56,57]. ID media was prepared by first growing cells in FBS-supplemented media for 24 h. The cells were then incubated in serum-free media overnight and then with the indicated treatments for 72 h. The media were next collected and depleted from proBDNF using anti-proBDNF antibodies. These ID media were then carefully removed and analyzed for the presence of proBDNF by ELISA (Biosensis). Significant depletion (95–100%) was observed upon using the antibodies employed in this study. The same amount of protein of each sample was analyzed in the experiments.

### 4.11. Western Blotting

Samples of the cell lysates or media collected as indicated were analyzed according to our previous protocols [44,67,69]. Attached live cells were harvested and the cell pellet was resuspended in 1 mL lysis buffer consisting of 20 mM Tris/HCl, pH 7.5, 137 mM NaCl, 1% triton X-100, 10% glycerol. Samples were briefly sonicated and centrifuged, and the supernatants were stored at −80 °C until further analysis. The protein concentrations were determined using the BCA protein assay kit. Following methods we reported previously [67], samples were boiled in 1X SDS, loaded and separated by SDS-PAGE on a 12% gel then transferred to a nitrocellulose membrane. The membrane was blocked in TBST buffer, pH 7.6, containing 5% nonfat milk for 6 h at 4 °C. The membrane was then incubated with the specific primary antibody in the blocking buffer, diluted as specified by the manufacturer at RT overnight with gentle shaking. After washing three times with TBST, the membrane was incubated with an HRP-linked secondary antibody in the blocking buffer, diluted according to the manufacturer’s recommendation. Subsequent to washing three times in TBST, the blots were developed using super signal west pico luminol (chemiluminescence) reagent and imaged with a Bio-Rad molecular imager.

### 4.12. siRNA Transfection

Transfections were carried out according to our methods reported earlier [69,73]. The day before transfection, cells were seeded at a density of 2 × 10^4^ cells in 25 cm^2^ flasks. Control siRNA, p53 siRNA, MMP2 or MMP9 siRNA were each mixed with Lipofectamine 2000 transfection reagent diluted in Opti-MEM Media (ThermoFisher) for 20 min at RT. The mixtures were then added to the cells at a final concentration of 100 nM for each siRNA, and the cells were incubated at 37 °C for 12 h followed by the specific treatments as indicated. The cells were then allowed to incubate from 24 to 72 h at 37 °C. Cells exposed to Lipofectamine 2000 alone were used as a mock control. The media were used to quantitate proBDNF and BDNF levels as described above. Cells collected by trypsinization at the different intervals after transfection were used for Western blotting, while cell viability and apoptosis were measured as described above. Each measurement represents the mean ± S.D. of 3–5 independent experiments, each performed in triplicate.

### 4.13. Statistical Analysis

The analysis was carried out as we previously reported [44,56,68,69]. Each experiment in this study was performed in triplicate and repeated a minimum of three times. Statistical values are expressed as the mean ± standard deviation (SD). To evaluate the statistical differences, the Mann–Whitney or an ordinary one-way ANOVA followed by Tukey’s post hoc multiple comparison test was performed. All the statistical tests were two-sided, and a *p* value of <0.05 was considered statistically significant in all cases. GraphPad Prism (GraphPad Software, 9.0.2) was used for the statistical analysis.

## Data Availability

Not applicable.
